# Preparation, Characterization and Wound Healing Effects of New Membranes Based on Chitosan, Hyaluronic Acid and Arginine Derivatives

**DOI:** 10.3390/polym10060607

**Published:** 2018-06-02

**Authors:** Andreea-Teodora Iacob, Maria Drăgan, Nicolae Ghețu, Dragoș Pieptu, Cornelia Vasile, Frédéric Buron, Sylvain Routier, Simona Elena Giusca, Irina-Draga Caruntu, Lenuța Profire

**Affiliations:** 1 Department of Pharmaceutical Chemistry, Faculty of Pharmacy, “Grigore T. Popa” University of Medicine and Pharmacy, 16 University Street, Iasi 700115, Romania; panzariu.andreea.teodora@gmail.com; 2 Department of Pharmaceutical Biotechnologies and Drug Industry, Faculty of Pharmacy, “Grigore T. Popa” University of Medicine and Pharmacy, 16 University Street, Iasi 700115, Romania; mwolszleger@yahoo.com; 3 Department of Plastic Surgery, Faculty of Medicine, “Grigore T. Popa” University of Medicine and Pharmacy, 16 University Street, Iasi 700115, Romania; ghetu.nicolae@umfiasi.ro (N.G.); dragos.pieptu@umfiasi.ro (D.P.); 4 Department of Physical Chemistry of Polymers, “Petru Poni” Institute of Macromolecular Chemistry, 41A Grigore GhicaVoda Alley, Iasi 700487, Romania; cvasile@icmpp.ro; 5 Institut de Chimie Organique et Analytique (ICOA), Univ Orleans, UMR CNRS 7311, F-45067 Orléans, France; frederic.buron@univ-orleans.fr (F.B.); sylvain.routier@univ-orleans.fr (S.R.); 6 Department of Morphofunctional Sciences, Faculty of Medicine, “Grigore T. Popa” University of Medicine and Pharmacy, 16 University Street, Iasi 700115, Romania; simona-eliza.giusca@umfiasi.ro (S.E.G.); irina.caruntu@umfiasi.ro (I.D.C.)

**Keywords:** chitosan, hyaluronic acid, arginine derivatives, wound dressing

## Abstract

New membranes based on chitosan and chitosan-hyaluronic acid containing new arginine derivatives with thiazolidine-4-one scaffold have been prepared using the ionic cross-linking method. The presence of the arginine derivatives with thiazolidine-4-one scaffold into the polymer matrix was proved by Fourier-transform infrared spectroscopy (FT-IR). The scanning electron microscopy (SEM) revealed a micro-porous structure that is an important characteristic for the treatment of burns, favoring the exudate absorption, the rate of colonization, the cell structure, and the angiogenesis process. The developed polymeric membranes also showed good swelling degree, improved hydrophilicity, and biocompatibility in terms of surface free energy components, which supports their application for tissue regeneration. Moreover, the chitosan-arginine derivatives (CS-6h, CS-6i) and chitosan-hyaluronic acid-arginine derivative (CS-HA-6h) membranes showed good healing effects on the burn wound model induced to rats. For these membranes a complete reepithelialization was observed after 15 days of the experiment, which supports a faster healing process.

## 1. Introduction

Skin and soft tissue injuries, such as burn, ulcer or other traumatic damages, represent a major health care problem in the entire world regarding the success of the therapy and the costs associated with it [1,2,3]. Although several wound dressings materials have been developed, the problem of wound management is far from being solved [4]. The big challenge of wound treatment remains promoting a faster wound healing and reducing the incidence of bacterial infection [5,6,7]. An ideal wound dressing material should have specific requirements such as: maintaining a moist environment and electrolyte balance at the wound interface, allowing gaseous exchange, removing excess of exudates [8], possessing antimicrobial properties for infection control, and promoting faster wound healing [9]. In addition, it should be easily available, inexpensive, non-allergic, and should also have hemostatic and analgesic properties [4].

Based on its specific characteristics, chitosan (CS) is one of the most important biopolymers for biomedical applications [10,11,12,13]. It is a hydrophilic high molecular weight cationic polysaccharide, biocompatible, safe, bioadhesive and biodegradable, derivative of chitin, extracted from the exoskeleton of shellfish [14,15,16]. Its low toxicity with versatile biological activities such as antibacterial and antifungal effects, hemostatic effects, low immunogenicity, scar prevention, and the ability to efficiently release drugs from the matrix, have provided ample opportunities for further development [17,18,19,20]. Chitosan and different materials based on chitosan have proven to possess favorable characteristics for promoting rapid dermal regeneration and accelerating wound healing [8,21,22]. 

Hyaluronic acid (HA) is another biopolymer with important biomedical applications including: tissue healing, increasing cell proliferation and migration, angiogenesis, as well as inflammatory response control [23]. It is a high molecular weight (10^4^–10^7^Da) non-sulfated glycosaminoglycan (GAG), which is a component of the extra cellular matrix (ECM) of many tissues, such as skin, synovial joints and periodontal tissues [24,25,26]. 

HA is involved in each stage of the wound healing process, including the inflammatory, granulation and reepithelialization stages [27,28]. It is also involved in the scavenging of reactive oxygen species (ROS) derived from polymorphonuclear leukocyte (PMN), which are strongly involved in the pathogenesisof wounds, especially chronic ones [29]. Based on its biological properties associated with its biocompatibility and biodegradability, many biomaterials derived from HA have been evaluated as potential biomedical devices [30,31,32,33,34]. 

In order to improve the physical and biological properties of these biopolymers, herein, we report the preparation, physic-chemical characterization, and biological evaluation of new chitosan and chitosan-hyaluronic acid based membranes that have incorporated new derivatives of arginine with thiazolidine-4-one scaffold as wound dressing biomaterials. It is known that arginine is a basic alpha-amino acid that has several pivotal roles in cellular physiology [35,36]. Arginine is the only precursor of nitric oxide (NO), a signal molecule involved in immune responses, angiogenesis, collagen synthesis, epithelialization, and the formation of granulation tissue, which are all essential processes for wound healing [37]. Based on the physiological roles of this aminoacid, new arginine derivatives with thiazolidine-4-one scaffold with improved antimicrobial and antioxidant effects have been developed by our research group [38].

## 2. Materials and Methods 

### 2.1. Reagents

Chitosan medium molecular weight (CS, *M_w_* = 425 kDa, 85% of degree of deacetylation and pKa ≈ 6.7), hyaluronic acid (HA, *M_w_* = 120kDa), *N^ω^*-nitro-l-arginine methyl ester hydrochloride (NO_2_-Arg-OMe, ≥98%, *M_w_* = 269.69), hematoxylin solution, Mayer’s (pH = 2.4), and eosin Y (dye content ≈99%) were purchased from Sigma Aldrich, Darmstadt, Germany. All other chemicals and reagents were of analytical grade and used without further purification.

### 2.2. Synthesis of Arginine Derivatives 

**Figure 1:**
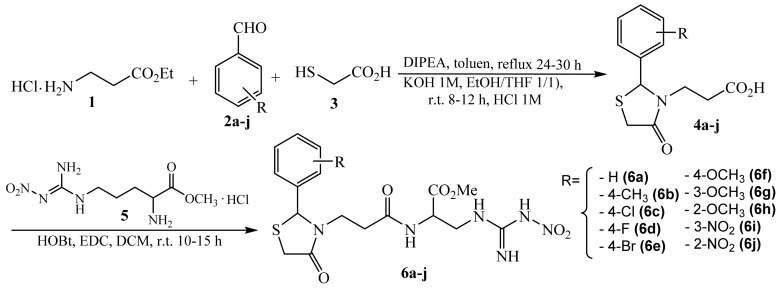
The synthesis of arginine derivatives with thiazolidine-4-one scaffold (ArgD: **6a**–**j**).

Starting from *N^ω^*-nitro-l-arginine methyl ester (NO_2_-Arg-OMe) hydrochloride, new arginine derivatives with thiazolidine-4-one scaffold were obtained according to the procedure described in our previous paper [38]. Briefly, the synthesis was performed in two steps (Figure 1). The first step consisted in the formation of the 1,3-thiazolidine-4-one heterocycle via one-pot condensation using ethyl 3-aminopropionate hydrochloride (**1**), aromatic aldehydes (**2a**–**j**), and thioglycolic acid (**3**). The resulted ethyl esters were reacted with potassium hydroxide 1M and then with hydrochloric acid 1M, resulting in the corresponding acid derivatives (**4a**–**j**). In the second step, the new arginine derivatives (**6a**–**j**) were obtained by condensation between acid derivatives (**4a**–**j**) and *N^ω^*-nitro-l-arginine methyl ester hydrochloride (**5**) in the presence of 1-ethyl-3-(3-dimethylaminopropyl) carbodiimide hydrochloride (EDC) and 1-hydroxybenzotriazole (HOBt) via amide bond formation.

### 2.3. Preparation of Chitosan—Arginine Derivatives (CS-ArgD) Membranes 

Chitosan medium molecular weight (CS, 2 g) was dissolved in 100 mL of 1% acetic acid by stirring at room temperature for 6 h [39]. The arginine derivatives (ArgD, **6a**–**j**) were added in concentrations of 1% (*w*/*v*) to the chitosan solution. The resulting blends were stirred for 24–48 h at room temperature and then 5 mL of each blend was poured into plastic Petri dishes (3 cm × 3 cm). That means into each Petri dish and into each sponge respectively, there is 50 mg of arginine derivatives. After freezing and freeze drying, the CS-ArgD membranes were crosslinked by immersion for 1 h at room temperature in 1% (*w*/*v*) solution of pentasodium tripolyphosphate (TPP). The excess of TPP was removed by washing several times with double-distilled water, and then the CS-ArgD membranes were frozen and freeze dried again for 12 h. Using a similar procedure a membrane containing *N^ω^*-nitro-l-arginine methyl ester (NO_2_-Arg-OMe) hydrochloride (CS-PArg), used as a reference membrane, has been developed. 

### 2.4. Preparation of Chitosan-Hyaluronic Acid-Arginine Derivatives (CS-HA-ArgD) Membranes 

Chitosan medium molecular weight (CS, 2 g) was dissolved in 100 mL of 1% acetic acid by stirring at room temperature for 6 h. The chitosan solution was neutralized with sodium hydroxide (NaOH) 1M and a hydrogel was obtained. Separately, hyaluronic acid (HA, 1 g) was dissolved in 100 mL of water by stirring for 12 h at room temperature. The two polymeric solutions were mixed in a ratio of 2:1 (chitosan:hyaluronic acid) and stirred for 12 h at room temperature [40]. To the resulted polymer blend, the arginine derivatives (ArgD, **6a**–**j**) were added in a concentration of 1% (*w*/*v*); they were stirred again for 24–48 h at room temperature and 5 mL of each blend was poured into plastic Petri dishes (3 cm × 3 cm). After freezing and freeze drying, the CS-HA-ArgD membranes were crosslinked by immersion for 1 h at room temperature in 1% (*w*/*v*) solution of pentasodium tripolyphosphate (TPP). The excess of TPP was removed by washing several times with double-distilled water, and then the CS-HA-ArgD membranes were frozen and freeze dried again for 12 h. Using a similar procedure, a membrane containing *N^ω^*-nitro-l-arginine methyl ester (NO_2_-Arg-OMe) hydrochloride (CS-HA-ArgD), used as a reference membrane, has been developed.

### 2.5. Characterization of Chitosan/Chitosan-Hyaluronic Acid-Arginine Derivatives (CS-ArgD, CS-HA-ArgD) Membranes

#### 2.5.1. Fourier-Transform Infrared Spectroscopy (FT-IR)

The presence of the arginine derivatives with thiazolidine-4-one scaffold into the polymer matrix was proved by FT-IR spectroscopy. The IR spectra were recorded using a ABB-MB 3000 FT-IR MIRacle^TM^ Single Bounce ATR-crystal ZnSe Fourier transform spectrometer, Quebec, QC, Canada, in the range of 4000–500 cm^−1^, at a resolution of 4 cm^−1^ using a total of 16 scans. The spectra were interpreted using Horizon program MB™ FT-IR.

#### 2.5.2. Morphology

The surface and morphology of the CS-ArgD and CS-HA-ArgD membranes were analyzed using a Fei Quanta 200F (field emission gun) scanning electron microscope (SEM), Hillsboro, OR, USA. The dried samples were coated with gold in order to create a conductive layer of metal which inhibits charging, reduces thermal damage and improves the secondary electron signal required for the examination in the SEM.

#### 2.5.3. Porosity Test 

The porosity degree of the CS-ArgD and CS-HA-ArgD membranes was determined using the method of immersing in ethanol 100% (*v*/*v*) at room temperature (20 °C). The membrane samples were weighed before immersion to determine the initial weight, after which they were immersed into ethanol 100% (*v*/*v*). After 24 h, the samples were weighed again and the porosity degree (P) was calculated based on the amount of ethanol absorbed by the membranes, using the following formula (1) [41]:

P (%) = (*W*_2_ − *W*_1_)/ρ*V* × 100 (1)

where: *W*_1_ is the weight of the dry membrane and *W*_2_ is the weight of the wet membrane, ρ is the density of the ethanol 100% (*v*/*v*) at room temperature and *V* is the volume of the wet membrane. The experiment was performed in triplicate for each sample.

#### 2.5.4. Swelling Ratio

The swelling degree of the CS-ArgD and CS-HA-ArgD membranes was determined by immersion in the phosphate buffer solution (PBS, pH 7.4) and sodium acetate buffer solution (pH 5) [11]. The membrane samples were weighed before immersion to determine the initial weight (*W*_0_), after which they were immersed into the buffer at room temperature (20 °C). Every 15 min, the samples were taken out from the buffer, wiped quickly with filter paper, and then weighed to determine the weight of the wet sample (*W_t_*). The operation was repeated until the thermodynamic equilibrium was reached. The membrane swelling ratio (MSR) was calculated using the following formula (2) [42]:

MSR (%) = (*W_t_* − *W*_0_)/*W*_0_ × 100 (2)

where: *W*_0_ is the weight of the dry membrane and *W_t_* is the weight of the wet membrane at different times. The experiment was performed in triplicate for each sample.

#### 2.5.5. Contact Angle Measurements

In order to perform the contact angle measurements, thin films were prepared. A sample of 200 µL of arginine derivatives-polymer blend (CS-ArgD, CS-HA-ArgD) prepared according to the procedure described in Section 2.3 and Section 2.4, was cast onto special glass surfaces (blades) and the samples were allowed to dry at room temperature. The contact angle was measured by the sessile drop method using CAM-200 equipment, KSV NIMA – Biolin Scientific, Espoo, Finland. 1 µL of pure liquid was placed on the film surface and the contact angle values were recorded within 10s, at room temperature and controlled humidity. The measurements were performed at least 10 times on different sites of the surface and the average value was considered [43,44].

#### 2.5.6. Surface Tension Parameters 

Based on the van Oss and Good acid-base method [45], the surface tension parameters were calculated, which divides the total surface tension into the dispersive Lifshitz–van der Waals interaction ($\gamma_{s}^{LW}$) and polar Lewis acid–base interactions ($\gamma_{s}^{AB}$), which are also subdivided into electron donor $\gamma_{s}^{-}$ (Lewis base) and electron acceptor $\gamma_{s}^{+}$ (Lewis acid) parts:

(3) $$\left(1\;+\;\cos\theta )\gamma_{s}^{TOT}\;=\;2(\sqrt{\gamma_{s}^{LW}\gamma_{l}^{LW}}\;+\;\sqrt{\gamma_{s}^{+}\gamma_{l}^{-}}\;+\;\sqrt{\gamma_{s}^{-}\gamma_{l}^{+}}\right)$$

where: θ is the contact angle measured in different liquids (double-distilled water, formamide and diiodomethane), $\gamma_{S}^{TOT}$ is the liquid total surface tension, and $\gamma_{l}^{LW}$and$\gamma_{s}^{LW}$are the apolar Lifshitz–van der Waals components of the liquid and the solid, respectively, whereas $\sqrt{\gamma_{s}^{+}\gamma_{l}^{-}}$ and $\sqrt{\gamma_{s}^{-}\gamma_{l}^{+}}$ are the Lewis acid–base contributions of either the solid or the liquid phase as indicated by the subscripts. 

### 2.6. Biological Evaluation

#### 2.6.1. Wound Healing Assay

The study was performed using white adult males Wistar rats, weighing between 250–300 g and it was approved by the Animal Research Committee of the “Grigore T. Popa” University of Medicine and Pharmacy, Iasi, Romania (no. 17826/2016). The rats were anesthetized by inhalation of isoflurane 2L/min, after which the dorsal areas were shaved and two burns were induced by applying a high-pressure steam at 114°C for 2 s through controlled electro-valve. After the debridement of the burn, the rats were randomly divided in 7 groups (of 6 rats each) and the burn surface was covered with the following materials: standard gauze dressing (control, group 1); CS-ArgD membrane: CS-6j (group 2), CS-6i (group 3), CS-6h (group 4), and CS-HA-ArgD; membranes: CS-HA-6j (group 5), CS-HA-6i (group 6), and CS-HA-6h (group 7). During the experiment the membranes were replaced on the 5th and 10th day in order to perform a complete and faster healing. At the time of replacing the membranes the wound was clean of damaged tissue, fibrin, dead cells, and excess of exudates. At every dressing renewal the burn surface was macroscopically analyzed and photographed and punch biopsies were performed. At the end of the experiment, on the 15th day, after the last punch biopsy, the rats were euthanized by intracardiac administration of 1–2 ccKCl under isoflurane anesthesia. The skin biopsies have been fixed, processed, paraffin embedded, and sectioned. To analyze the wound healing results Hematoxylin and Eosin (H&E) staining was used. The analysis of the biopsy samples was performed using a Leica DM3000 microscope, Leica Microsystems, Wetzlar, Germany, including a special module for analysis and image processing. 

#### 2.6.2. Statistical Analysis

The data are expressed as mean ± standard deviation (SD). The statistical software package StatView was used for the analysis of biological results. The experimental data were analyzed by repeated measures by 3 (groups) × 3 (time sample points), using Analysis of Variance (ANOVA) and Fisher’s post hoc test to compare the burn surface area between groups at Day 5, 10 and 15. Statistical significance was set to *p* value ≤ 0.05.

## 3. Results

### 3.1. Chemistry

The synthesis of arginine derivatives with thiazolidine-4-one scaffold (ArgD: **6a**–**j**) is summarized in Figure 1. The procedure and full spectral characterization of the compounds (FT-IR, ^1^H-RMN, ^13^C-RMN, MS) are presented in our previous paper [38].

The difference between synthesized compounds consists of the radical that substitutes the aromatic ring from C_2_ of the thiazolidinel-4-one moiety. The substitution pattern of the aromatic ring was carefully selected, using both electron donating groups such as –CH_3_, –OCH_3_, and –OH, in addition to electron withdrawing groups such as halogens (Cl, F, Br) and –NO_2_, in order to improve the biological effects. There are some studies that have proved that the substitution of the aromatic ring cu halogens has a positive influence on the antibacterial effects of the compounds with thiazolidine-4-one structures [46]. 

### 3.2. Characterization of Chitosan/Chitosan-Hyaluronic Acid-Arginine Derivatives (CS-ArgD, CS-HA-ArgD) Membranes

#### 3.2.1. FT-IR Spectral Data

**Figure 2:**
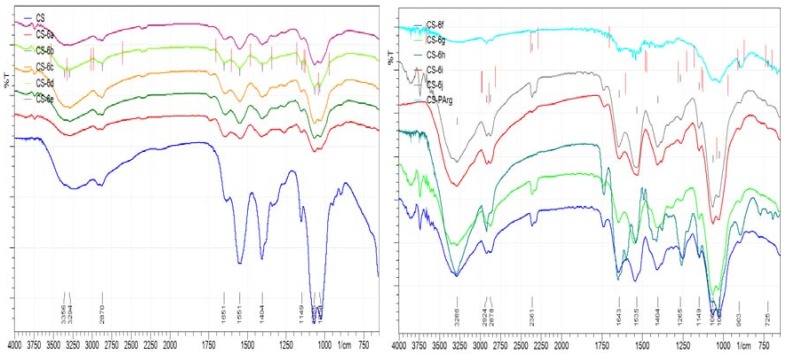

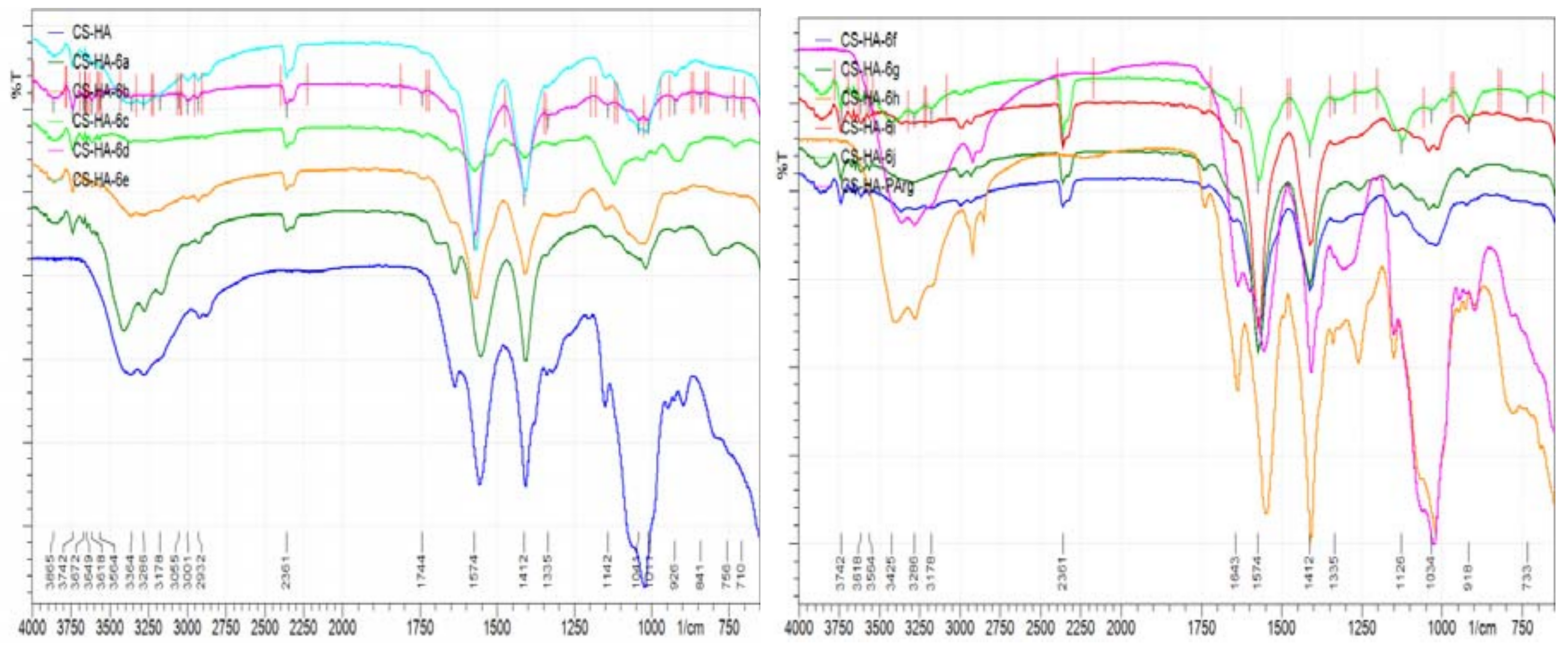
Infrared (IR) spectra of CS-ArgD and CS-HA-ArgD membranes.

The infrared spectra of the CS-ArgD and CS-HA-ArgD membranes revealed the characteristic absorption bands of the polymers (CS, HA) and also of the arginine derivatives (ArgD: **6a**–**j**) (Figure 2). Thus, for CS-ArgD membranes, the characteristic absorption bands of CS were identified as an abroad band between 3400–3600 cm^−1^ attributed to the stretching vibration of OH, NH_2_ and NH groups and a narrower absorption band around 3200 cm^−1^ assigned to the CH groups. These bands are more intense in the CS-HA-ArgD membranes due to the specific groups of HA. The amide group (–CO–NH–) of the polymers appears as a characteristic band at 1550–1600 cm^−1^ (CS-ArgD) and at 1490–1550 cm^−1^ (CS-HA-ArgD). The band from 1300–1400 cm^−1^ is attributed to the carboxyl group of HA. The presence of the arginine derivatives (**6a**–**j**) in the polymer matrix was proven by the specific aromatic ring absorption bands, which appear within the 3100–2954 cm^−1^ range and by the methylene group absorption band, which appears at the wavelength of 1250 cm^−1^.

#### 3.2.2. Morphology

**Figure 3:**
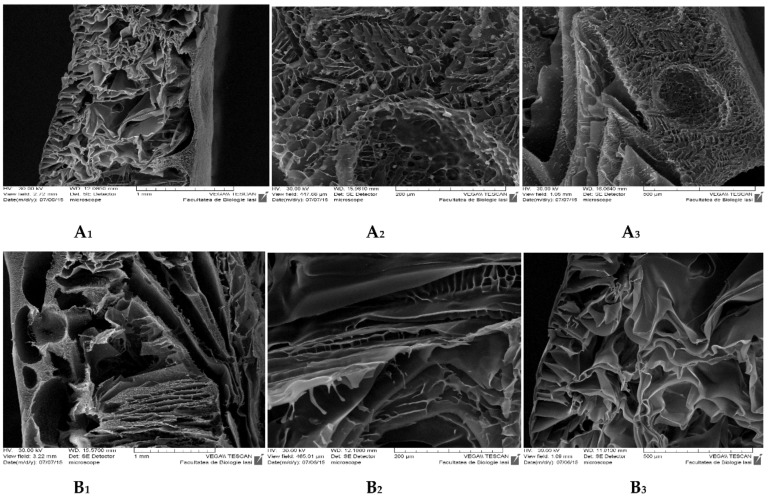
Scanning electron microscope (SEM) images for CS-PArg (**A_1_**–**A_3_**) and CS-6h (**B_1_**–**B_3_**) membranes at various magnifications of 1 mm, 200 µm, 500 µm.

The SEM analysis indicates that, in most of the cases, the incorporation of ArgD (**6a**–**j**) into the chitosan and chitosan-hyaluronic acid matrix results in an increasing of the pores’ size of the polymeric membrane, which could be explained by the presence of the thiazolidine-4-one scaffold and of the arginine structure. This feature is particularly important regarding the application of the CS-ArgD and CS-HA-ArgD membranes in the treatment of burns, favoring the exudates absorption from the wound. In Figure 3 there are presented the SEM images for CS-6h membrane (**6h**: *N*^2^-[(2-(4-methoxyphenyl)-4-oxo-1,3-thiazolidin-3-yl)propionyl]-nitro-l-arginine methyl ester) (B) for which it was observed the biggest porosity in comparison to CS-PArg (A), which contains NO_2_-Arg-OMe, which is used as a starting material in the synthesis of arginine derivatives. 

#### 3.2.3. Porosity Test

**Figure 4:**
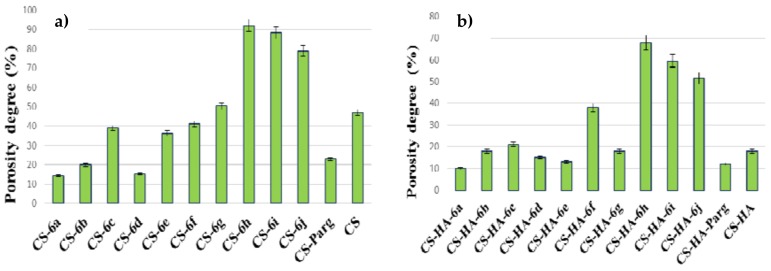
The porosity degree of the CS-ArgD (**a**) and CS-HA-ArgD (**b**) membranes.

The porosity degree is an important characteristic of polymeric membranes used in the treatment of wounds, since it influences the exudates absorption, the rate of colonization, the cell structure and the angiogenesis process. It was shown that chitosan membranes with smaller pore sizes are characterized by an improved mechanical strength, good water absorption rate and increased cellular effects, unlike the chitosan membranes with larger pores [41]. At the same time the beneficial effects of membranes with high porosity structure for wound healing were demonstrated because they facilitate the transport of nutrients and oxygen and the absorption of wound exudates [39]. The porosity degree depends on the type and concentration of the polymer and the characteristics of the drug loaded into the polymer matrix, on the freezing method, as well as on the crosslinking agent used. In our study it was observed that the incorporation of arginine derivatives with thiazolidine-4-one scaffold (**6a**–**j**) into the chitosan matrix was associated, in most of the cases, with increasing the porosity degree in comparison with the CS-PArg that contains the NO_2_-Arg-OMe. Moreover, in several cases the porosity degree of CS-ArgD was higher than the porosity degree of the chitosan membrane (CS) (Figure 4a). Regarding the influence of the structure of ArgD incorporated into the polymer matrix on the degree of porosity, it was observed that nitro (*ortho* and *meta*) and methoxy (*ortho*) from the aromatic ring from the C_2_ of the thiazolidinel-4-one moiety increase the porosity degree. The highest porosity degree was showed by CS-6h (R = 2-OCH_3_, P = 92.05%), which was two times higher than the value recorded for the chitosan matrix (P = 47.02%). A good porosity degree, higher than the chitosan matrix, was also showed by CS-6i (R = 3-NO_2_, P = 88.25%) and CS-6j (R = 2-NO_2_, P = 78.87%).

In the case of CS-HA-ArgD membranes, the porosity degree ranged from 10% to 70% (Figure 4b). The highest porosity degree was showed by CS-HA-6h (R = 2-OCH_3_, P = 67.80%), CS-HA-6i (R = 3-NO_2_, P = 59.50%) and CS-HS-6j (R = 2-NO_2_, P = 51.40%). With few exceptions, the CS-HA-ArgD membranes showed a lower degree of porosity compared to the corresponding CS-ArgD matrices, which means that the hyaluronic acid present in the polymer matrix is responsible for this characteristic.

#### 3.2.4. Swelling Degree

**Figure 5:**
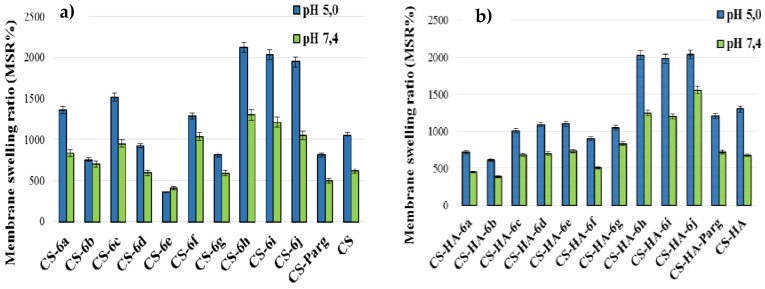
The membrane sweeling ratio (MSR) (%) of CS-ArgD (**a**) and CS-HA-ArgD (**b**) membranes.

The swelling degree was studied at pH 7.4, similar to the physiological one and at pH 5, similar to the skin tissue injuries [47]. The data showed that the swelling degree of the CS-ArgD and CS-HA-ArgD membranes was higher at pH 5 than at pH 7.4, especially for CS-ArgD (Figure 5). This is a very important characteristic because it means that they have a good absorption capacity of exudates in the wound healing process. The higher degree of swelling recorded at pH 5 can be explained by the protonated amino groups. It is known that chitosan has a weak basic character with a pKa around 6.5 and its swelling capacity is largely due to its free amino groups. In the acid environment the amino groups are positively charged and induce repulsive forces in the polymer matrix and as a result the network volume is increasing. It is expected that the membranes with a greater degree of porosity will also have a higher MSR, a fact confirmed by the results obtained. The highest swelling degree was recorded for the same derivatives that recorded an increased porosity degree: CS-6h (R = 2-OCH_3_, MSR = 2124%) and CS-6i (R = 3-NO_2_, MSR = 2033%) at pH 5, which was approximately two times higher than that of CS (MSR=1054%). A higher swelling degree than chitosan was also recorded for CS-6j (R = 2-NO_2_, MSR = 1940%). All CS-ArgD membranes showed a higher MSR than CS-PArg membrane, which contains the parent arginine derivative (NO_2_-Arg-OMe) (Figure 5a), which persisted for 24 h.

In the case of the CS-HA-ArgD membranes, the highest values of the swelling degree were recorded for CS-HA-6j (R = 2-NO_2_, MSR = 2036%), CS-HA-6h (R = 2-OCH_3_, MSR = 2030%), CS-HA-6i (R = 3-NO_2_, MSR = 1979%), and CS-HA-6d (R = 4-F, MSR = 1979%) at pH 5, values which were higher than the value recorded for the CS-HA matrix (MSR = 1320%) (Figure 5b). In was also noted that the thermodynamic equilibrium had been reached after 120 min for CS-ArgD and faster, after 60 min, for CS-HA-ArgD, which is explained by the hydrophilic properties of the hyaluronic acid. 

#### 3.2.5. Surface Tension Parameters 

One method to evaluate the degree of hydrophilicity and biocompatibility of the polymeric materials is to determine the surface tension parameters. For tissue engineering applications it is necessary to have an appropriate balance of hydrophilic and hydrophobic surface features, because it is known that excessively hydrophobic surfaces enhance the cell affinity and reduce biocompatibility, while highly hydrophilic surfaces reduce the cell-cell interactions [48]. The surface tension expresses the intramolecular interactions, which are produced on a biomaterial surface, having implications on the cell adhesion process and on the biomedical application of polymers. There is a strong connection between the surface tension and the cell adhesion. It has been shown that the cell adhesion is increasing with increasing surface tension [49] and with increasing wettability of the membrane [48]. 

In our study, the surface tension parameters of CS-ArgD and CD-HA-ArgD films were estimated based on contact angle measurements using three pure liquids: double-distilled water, formamide, and diiodomethane.

**Figure 6:**
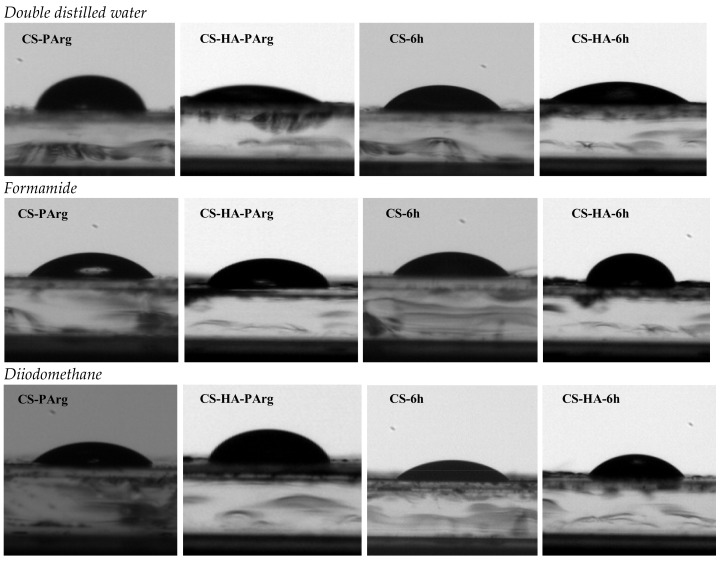
Recorded images at the contact angle measurements for CS-6f/CS-HA-6h and CS-PArg/CS-HA-PArg in double distilled water, formamide, and diiodmethane.

In the case of CS-ArgD films, it was observed that the incorporation of arginine derivatives with thizolidine-4-one scaffold (**6a**–**j**) resulted in the decreasing of the value of the contact angle compared to the CS and CS-NO_2_-Arg-OMe (CS-PArg) films. It can be noted that all CS-ArgD films are hydrophilic because the value of the contact angle is less than 90°. The most hydrophilic are the CS-6a (R = H) and CS-6d (R = 4-F) films, for which the values of the contact angle were 50.88 ± 0.9 and 57.84 ± 1.5, and the less hydrophilic film is the CS-6h (R = 2-OCH_3_) where the contact angle recorded was 85.90 ± 3.5 (Table 1, Figure 6). 

The presence of the hyaluronic acid in the polymeric matrix substantially increases the hydrophilicity; for example, all of the CS-HA-ArgD films were more hydrophilic than the corresponding CS-ArgD films. The value of the contact angle measured in double distilled water ranged between 16.07 ± 0.5 (CS-HA-6a) and 47.67 ± 1.9 (CS-HA-6h) (Table 1, Figure 6). 

In addition, two other measurements of the contact angle, in diiodomethane and formamide, have been performed (Table 1), in order to evaluate the surface tension parameters. The values are presented in Table 2. For the CS-ArgD films it was observed that the presence of arginine derivatives with tiazolidine-4-one scaffold (**6a**–**j**) into the chitosan matrix slightly increased the value of total surface tension ($\gamma_{s}^{TOT}$), especially when compared to the value recorded for the chitosan matrix. However, the values recorded for CS-ArgD were lower than the ones for the CS-PArg membrane, which means that the modulation of the arginine structure using the thiazolidine-4-one scaffold has a beneficial effect on the surface tension parameters and thus on the biocompatibility of the membranes. Referring to the free energy polar component (*γ^AB^*), it can be estimated that by incorporating the arginine derivatives (**6a**–**j**) the polarity of the chitosan increases. Moreover, the free energy surface (*γ^EF^*) is higher than 15 mN/m, which means that the CS-ArgD films have a procoagulant effect, which can be useful regarding their potential application in the management of burns. This effect is supported, by the known hemostatic effects of chitosan [8].

For the CS-HA-ArgD films it was observed that, in most cases, the values of total surface tension *(*$\gamma_{s}^{TOT}$*)* were similar to the values recorded for the CS-HA matrix (56.21mN/m), which means that the incorporation of the arginine derivatives with the thiazolidine-4-one scaffold did not reduce the biocompatibility of the CS-HA matrix. 

For this type of film it was also observed that the values of the free energy surface ($\gamma^{EF}$) are lower than the values calculated for the CS-ArgD films, which is explained by the presence of the hyaluronic acid in the polymer matrix.

### 3.3. Biological Evaluation

#### Wound Healing Assay

For the wound healing assay, six membranes were selected for testing; three CS-ArgD (CS-6h, CS-6i, CS-6j) and their corresponding CS-HA-ArgD. The selection was based on the biological evaluation of the arginine derivatives with thiazolidine-4-one scaffold (**6a**–**j**), as well as on the characteristics of the polymeric membranes. The compounds **6h**, **6i** and **6j** showed good antimicrobial and antioxidant effects—properties which are very important in the healing process. The results were presented in our previous paper [38]. Moreover, the selected polymer matrices showed the best porosity and swelling degree, which are important characteristics for topical use in the treatment of injuries caused by burns.

**Figure 7:**
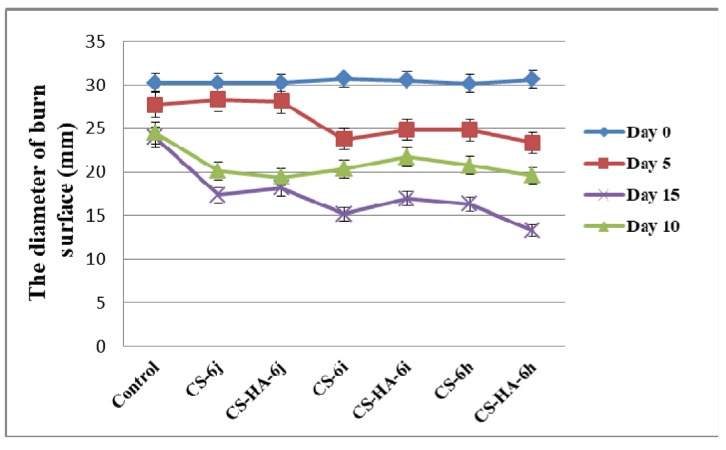
The diameter of burn surface (mm) at different times (CS-chitosan, HA-hyaluronic acid).

**Figure 8:**
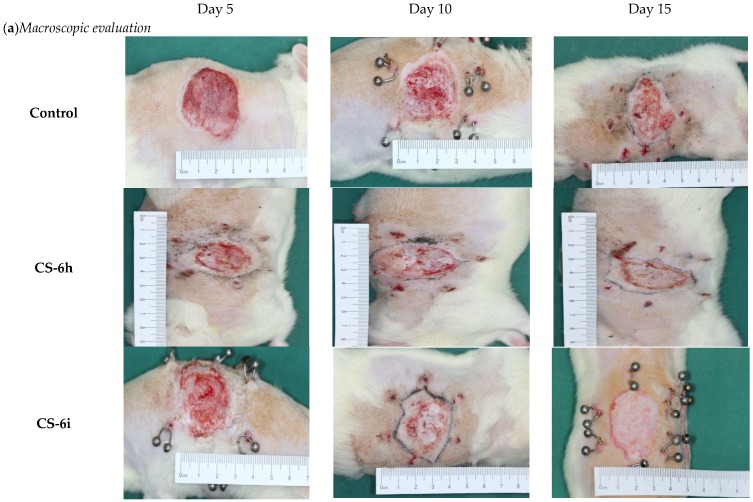

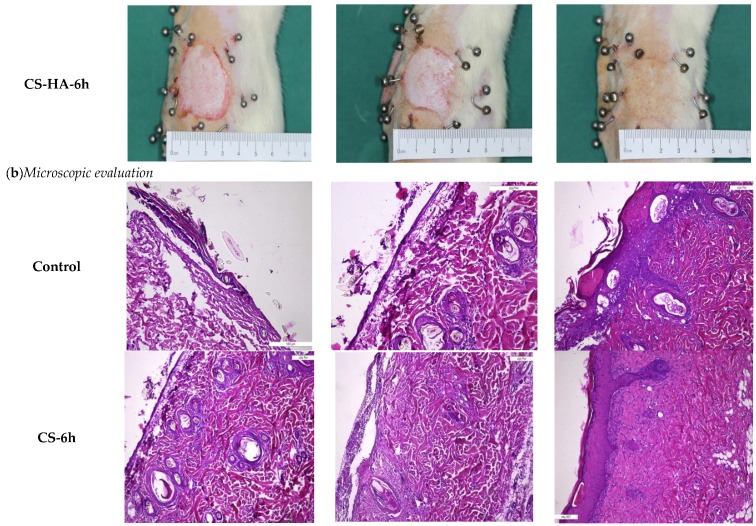

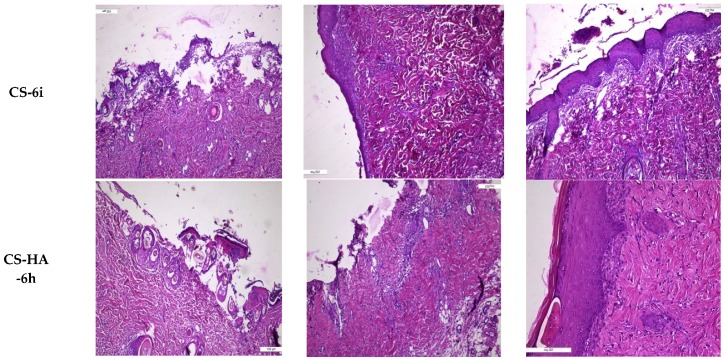
Macroscopic (**a**) and microscopic (**b**) evaluation of burn wound area of the polymer membranes and control at various timelines: on the 5th, 10th and 15th day of the experiment.

The macroscopic evaluation of the healing effects of the polymer membranes was carried by measuring the diameter of the wound area at different timelines (on the 5th, 10th and 15th day), in comparison to the initial diameter of the burned area (day 0) (Figure 7). The burn reduction was more intense for all groups treated with polymer membranes, compared to the control group treated with standard gauze dressing. Compared to the control (23.96 ± 1.11 mm), the best results at the end of the experiment were obtained for the groups treated with CS-6h (16.34 ± 0.54 mm), CS-6i (15.19 ± 0.36 mm), and CS-HA-6h (13.26 ± 0.23 mm). For the control group, the burn is characterized on the 5th and 10th day of the experiment by hemorrhagic or sero-citrus blisters and, in some cases, even by deepening wound suppurations. At the end of the experiment, the burn is slightly pearly with reduced signs of healing. For the groups treated with polymer membranes, the burn does not have suppuration areas, its appearance is rosacea and it shows a good blood supply. In addition, on the 10th and 15th day of the experiment, glossy areas can be observed, which represent the new layer of epithelial cells as a result of the tissue regeneration process. In Figure 8a there are presented macroscopic images for the groups treated with the control and with CS-HA-6j.

The microscopic evaluation showed that the best results were obtained for the groups treated with CS-6h, CS-6i, and CS-HA-6h (Figure 8b). On the 5th day of the experiment, there was observed extended epithelial denudation and small isolated areas of reepithelialization in continuity with the epithelial sheaths of the hair follicles, while on the 10th day of the experiment there were observed epithelial regeneration aspects with an epidermis that showed a reduced thickness. At the end of the experiment it was observed a complete epithelialization: the epidermis with different sizes (5–20 cell lines), showing incomplete cell maturation of spinous layer, and a compact stratum corneum. Dermo-epidermal interfaces with a tendency to curl and a superficial dermis presenting active fibroblasts and congestive capillaries were also observed (Figure 8b). 

## 4. Conclusions

New chitosan and chitosan-hyaluronic acid membranes containing novel arginine derivatives with thiazolidine-4-one scaffold have been developed as potential wound dressing materials. These polymeric membranes have been characterized to evaluate the structure and morphology, the porosity degree, the swelling capacity, the hydrophilicity and surface tension parameters. For the developed membranes, the degree of porosity, the hydrophilicity and the swelling capacity were higher than for the membrane containing the parent arginine derivative (NO_2_-Arg-OMe). Moreover, the chitosan-6h (CS-6h), chitosan-6i (CS-6i) and chitosan-hyaluronic acid-6h (CS-HA-6h) membranes showed good healing effects on a burn wound model. For these membranes, the reepithelialization was completed with lower values of a damaged tissue zone diameter compared to the control, which supports a faster healing process. All of the correlated data of this study show that the developed novel chitosan-arginine derivatives and the chitosan-hyaluronic acid-arginine derivatives membranes are potential materials for wound dressing, with evident beneficial effects in burn wound healing.

## Figures and Tables

**Table 1:** The contact angle values recorded for CS-ArgD and CS-HA-ArgD films.

CS-ArgD/CS-HA-ArgD	Contact Angle Value (°)					
	Double-Distilled Water		Formamide		Diiodomethane	
	CS	CS-HA	CS	CS-HA	CS	CS-HA
**CS/CS-HA**	102.14 ± 2.3	10.97 ± 1.9	69.86 ± 4.5	40.23 ± 2.2	65.16 ± 0.2	89.98 ± 1.1
**NO_2_-Arg-OMe**	102.52 ± 3.1	26.76 ± 2.3	47.77 ± 0.7	39.8 ± 1.9	54.00 ± 4.7	88.97 ± 0.5
**6a**	50.88 ± 0.9	16.07 ± 0.5	47.79 ± 1.5	12.34 ± 2.6	41.99 ± 1.0	40.99 ± 3.8
**6b**	79.07 ± 1.7	35.34 ± 4.6	56.44 ± 2.3	30.27 ± 3.5	47.68 ± 0.8	67.54 ± 2.9
**6c**	77.17 ± 4.3	16.07 ± 0.5	51.83 ± 3.5	35.41 ± 0.7	46.81 ± 2.7	58.66 ± 1.7
**6d**	57.84 ± 1.5	30.39 ± 2.1	39.22 ± 0.6	34.09 ± 4.3	43.77 ± 3.1	66.43 ± 1.6
**6e**	74.88 ± 1.4	26.74 ± 3.0	59.50 ± 2.8	23.29 ± 0.8	46.87 ± 4.3	70.89 ± 2.3
**6f**	71.35 ± 0.3	36.21 ± 2.9	55.93 ± 2.0	46.56 ± 1.6	39.86 ± 4.4	56.78 ± 3.3
**6g**	73.15 ± 2.6	32.17 ± 0.3	59.79 ± 4.3	24.98 ± 1.8	44.45 ± 1.7	68.79 ± 0.7
**6h**	85.90 ± 3.5	47.67 ± 1.9	62.21 ± 1.7	38.05 ± 4.1	53.93 ± 2.3	57.09 ± 4.4
**6i**	83.36 ± 1.2	40.96 ± 1.1	61.89 ± 0.3	26.37 ± 2.9	37.21 ± 0.8	60.54 ± 4.6
**6j**	84.98 ± 2.9	38.79 ± 3.8	54.78 ± 1.2	17.65 ± 2.5	51.18 ± 4.5	45.57 ± 2.4

**Table 2:** The surface tension parameters (mN/m) for CS-ArgD andfor CS-HA-ArgDfilms.

Films	The Surface Tension Parameters (mN/m)					
	$\gamma_{s}^{LW}$	$\gamma_{s}^{+}$	$\gamma_{s}^{-}$	$\gamma_{s}^{AB}$	$\gamma_{s}^{TOT}$	$\gamma_{s}^{FE}$
*CS-ArgDfilms*						
**CS**	25.56	1.52	0.04	0.52	26.08	31.31
**CS-PArg**	31.95	6.93	4.90	11.65	43.60	32.27
**CS-6a**	38.51	0.02	34.80	1.59	40.11	31.09
**CS-6b**	35.48	0.45	5.79	3.23	38.72	24.88
**CS-6c**	35.96	0.91	5.39	4.43	40.39	22.59
**CS-6d**	37.59	1.13	18.73	9.19	42.77	16.86
**CS-6e**	35.93	0.35	6.00	2.04	37.96	28.37
**CS-6f**	39.60	0.22	5.08	1.88	41.48	30.56
**CS-6g**	37.23	0.18	5.56	1.60	38.83	30.47
**CS-6h**	31.99	0.36	5.19	2.74	34.73	24.63
**CS-6i**	40.90	0.01	5.02	0.45	41.35	38.25
**CS-6j**	33.54	1.48	6.62	3.10	36.65	24.36
*CS-HA-ArgDfilms*						
**CS-HA**	12.72	6.68	70.81	43.49	56.21	0.35
**CS-HA-NO_2_-Arg-OMe**	13.13	8.71	44.74	39.49	52.62	0.13
**CS-HA-6a**	39.03	1.42	51.58	17.13	56.16	12.06
**CS-HA-6b**	24.21	4.79	40.78	27.96	52.17	3.05
**CS-HA-6c**	29.29	2.73	31.51	18.55	47.83	7.30
**CS-HA-6d**	24.84	4.11	36.92	24.62	49.46	3.86
**CS-HA-6e**	22.33	6.43	45.61	34.26	56.59	1.94
**CS-HA-6f**	30.36	7.08	92.86	31.14	91.50	7.81
**CS-HA-6g**	23.50	5.96	41.07	31.29	54.79	2.46
**CS-HA-6h**	30.19	1.42	44.30	15.88	46.06	8.87
**CS-HA-6i**	28.21	3.56	43.63	24.93	53.13	4.97
**CS-HA-6j**	36.63	2.07	42.33	18.71	55.34	10.29

$\gamma_{s}^{LW}$ = Lifshitz–van der Waals contributions; $\gamma_{s}^{+}$ = Lewis acid-base electron acceptor contributions; $\gamma_{s}^{-}$ = acid-base Lewis electron donor component; $\gamma_{s}^{AB}$ = polar Lewis acid-base component; $\gamma_{s}^{TOT}$ = total solid surface tension; $\gamma_{s}^{FE}$ = free surface energy.

## References

[1] Powers J.G., Higham C., Broussard K., Phillips T.J. (2016). Wound healing and treating wounds: Chronic wound care and management. J. Am. Acad. Dermatol..

[2] Okabayashi R., Nakamura M., Okabayashi T., Tanaka Y., Nagai A., Yamashita K. (2009). Efficacy of polarized hydroxyapatite and silk fibroin composite dressing gel on epidermal recovery from full-thickness skin wounds. J. Biomed. Mater. Res..

[3] Tavakoli J., Mirzaei S., Tang Y. (2018). Cost-effective double-layer hydrogel composites for wound dressing applications. Polymers.

[4] Bano I., Arshad M., Yasin T., Ghauri M.A. (2017). Chitosan: A potential biopolymer for wound management. Int. J. Biol. Macromol..

[5] Zhang D., Zhou W., Wei B., Wang X., Tang R., Nie J., Wang J. (2015). Carboxyl-modified poly(vinyl alcohol)-crosslinked chitosan hydrogel films for potential wound dressing. Carbohydr. Polym..

[6] Vowden K., Vowden P. (2017). Wound dressings: Principles and practice. Surgery.

[7] Pintilie L., Negut C., Oniscu C., Caproiu M.T., Nechifor M., Iancu L., Ghiciuc C., Ursu R. (2009). Synthesis and antibacterial activityof some novel quinolones. Rom. Biotechnol. Lett..

[8] Jayakumar R., Prabaharan M., Sudheesh Kumar P.T., Nair S.V., Tamura H. (2011). Biomaterials based on chitin and chitosan in wound dressing applications. Biotechnol. Adv..

[9] Xiao B., Wan Y.Y., Zhao M., Liu Y., Zhang S. (2011). Preparation and characterization of antimicrobial chitosan-N-arginine with different degrees of substitution. Carbohydr. Polym..

[10] Ye M., Lian X., Huaping T., Ming F., Jianliang L., Yang J., Zhonghua L., Yong C., Xiaohong H. (2017). Chitosan membrane dressings toughened by glycerol to load antibacterial drugs for wound healing. Mater. Sci. Eng..

[11] Nitta S., Kaketani S., Iwamoto H. (2015). Development of chitosan-nanofiber-based hydrogels exhibiting high mechanical strength and pH-responsive controlled release. Eur. Polym. J..

[12] Castro S.P.M., Lizárraga Paulín E.G. (2012). Chitosan a new panacea? Areas of application. The Complex World of Polysaccharides.

[13] Zhao D., Yu S., Sun B., Gao S., Guo S., Zhao K. (2018). Biomedical applications of chitosan and its derivative nanoparticles. Polymers.

[14] Lupascu F.G., Dash M., Samal S.K., Dubruel P., Lupușoru C.E., Lupusoru R.-V., Dragostin O., Profire L. (2015). Development, optimization and biological evaluation of chitosan scaffold formulations of new xanthine derivatives for treatment of type-2 diabetes mellitus. Eur. J. Pharm. Sci..

[15] Reys L.L., Silva S.S., Pirraco R.P., Marques A.P., Mano J.F., Silva T.H., Reis R.L. (2017). Influence of freezing temperature and deacetylation degree on the performance of freeze-dried chitosan scaffolds towards cartilage tissue engineering. Eur. Polym. J..

[16] Cheng S.Y., Wang B.J., Weng Y.M. (2015). Antioxidant and antimicrobial edible zein/chitosan composite films fabricated by incorporation of phenolic compounds and dicarboxylic acids. LWT Food Sci. Technol..

[17] Mercy H.P., Halim A.S., Hussein A.R. (2012). Chitosan-derivatives as hemostatic agents: Their role in tissue regeneration. Neural Regen. Res..

[18] Lee J., Duncan A., Townsend S., Baker S. (2014). Synthesis and characterization of a chitosan derivative 976.3. FASEB J..

[19] Wang X., Yan Y., Zhang R. (2006). A comparison of chitosan and collagen sponges as hemostatic dressings. J. Bioact. Compat. Polym..

[20] Goy R.C., Britto D., Assis O.B.G. (2009). A review of the antimicrobial activity of chitosan. Polimeros.

[21] Patrulea V., Ostafe V., Borchard G., Jordan O. (2015). Chitosan as a starting material for wound healing applications. Eur. J. Pharm. Biopharm..

[22] Dragostin O.M., Samal S.K., Dash M., Lupascu F., Pânzariu A., Tuchiluș C., Ghețu N., Danciu M., Dubruel P., Pieptu D. (2016). New antimicrobial chitosan derivatives for wound dressing applications. Carbohydr. Polym..

[23] Gyles D.A., Castro L.D., Carréra Silva J.O., Ribeiro-Costa R.M. (2017). A review of the designs and prominent biomedical advances of natural and synthetic hydrogel formulations. Eur. Polym. J..

[24] Necas J., Bartosikova L., Brauner P., Kolar J. (2008). Hyaluronic acid (hyaluronan): A review. Vet. Med..

[25] Mayet N., Choonara Y.E., Kumar P., Tomar L.K., Tyagi C., Du Toit L.C., Pillay V. (2014). A comprehensive review of advanced biopolymericwoundhealing systems. J. Pharm. Sci..

[26] Mero A., Campisi M. (2014). Hyaluronic acid bioconjugates for the delivery of bioactive molecules. Polymers.

[27] Mogosanu G.D., Grumezescu A.M. (2014). Natural and synthetic polymers forwoundsand burns dressing. Int. J. Pharm..

[28] Abdel-Basset Sanada R., Abdel-Bar H.M. (2017). Chitosan-hyaluronic acid composite sponge scaffold enriched with Andrographolide-loaded lipid nanoparticles for enhanced wound healing. Carbohydr. Polym..

[29] Mohandas A., Anisha B.S., Chennazhi K.P., Jayakumar R. (2015). Chitosan-hyaluronic acid/VEGF loaded fibrin nanoparticles composite sponges for enhancing angiogenesis in wounds. Colloids Surf. B Biointerfaces.

[30] Martínez-Sanz E., Ossipov D.A., Hilborn J., Larsson S., Jonsson K.B., Varghese O.P. (2011). Bone reservoir: Injectable hyaluronic acid hydrogel for minimal invasive bone augmentation. J. Control. Release.

[31] Bajaj G., Kim M.R., Mohammed S.I., Yeo Y. (2012). Hyaluronic acid-based hydrogel for regional delivery of paclitaxel to intraperitoneal tumors. J. Control. Release.

[32] Ling L., Ning W., Xun J., Deng R., Shihong N., Sun L., Qinjie W., Yuquan W., Changyang G. (2014). Biodegradable and injectable in situ cross-linking chitosan-hyaluronic acid based hydrogels for postoperative adhesion prevention. Biomaterials.

[33] Schantéa C.E., Zuber G., Herlin C., Vandamme T.F. (2011). Chemical modifications of hyaluronic acid for the synthesis of derivatives for a broad range of biomedical applications. Carbohydr. Polym..

[34] Iwasaki N., Kasahara Y., Yamane S., Igarashi T., Minami A., Nisimura S. (2011). Chitosan-based hyaluronic acid hybrid polymer fibers as a scaffold biomaterial for cartilage tissue engineering. Polymers.

[35] Gould A., Naidoo C., Candy G.P. (2008). Arginine metabolism and wound healing. Wound Heal. S. Afr..

[36] Tang H., Zhang P., Kieft T.L., Ryan S.J., Baker S.M., Wiesmann W.P., Rogelj S. (2010). Antibacterial action of a novel functionalized chitosan-arginine against Gram-negative bacteria. Acta Biomater..

[37] Debats I.B.J.G., Wolfs T.G.A.M., Gotoh T., Cleutjens J.P.M., Peutz-Kootstra C.J., van der Hulst R.R.W.J. (2009). Role of arginine in superficial wound healing in man. Nitric Oxide.

[38] Pânzariu A.T., Apotrosoaei M., Vasincu I.M., Drăgan M., Constantin S., Buron F., Routier S., Profire L., Tuchiluș C. (2016). Synthesis and biological evaluation of new 1,3-thiazolidine-4-one derivatives of nitro-L-arginine nethyl ester. Chem. Cent. J..

[39] Anisha B.S., Sankar D., Mohandas A., Chennazhi K.P., Nair S.V., Jayakumar R. (2013). Chitosan–hyaluronan/nano chondroitin sulfate ternary composite sponges for medical use. Carbohydr. Polym..

[40] Anisha B.S., Biswas R., Chennazhi K.P., Jayakumar R. (2013). Chitosan-hyaluronic acid/nano silver composite sponges for drug resistant bacteria infected diabetic wounds. Int. J. Biol. Macromol..

[41] Lua B., Lua F., Zou Y., Jiawei L., Rong B., Zhiquan L., Fangying D., Dayang W., Guangqian L. (2017). In situ reduction of silver nanoparticles by chitosan-l-glutamic acid/hyaluronic acid: Enhancing antimicrobial and wound-healing activity. Carbohydr. Polym..

[42] Lin Y.C., Tan F., Marra K.G., Jan S.S., Liu D.C. (2009). Synthesis and characterization of collagen/hyaluronan/chitosan composite sponges for potential biomedical applications. Acta Biomater..

[43] Rotta J., Ozório R.A., Kehrwald A.M., Mariz de Oliveira Barra G., Dias de Melo Castanho Amboni R., Barreto P.L.M. (2009). Parameters of color, transparency, water solubility, wettability and surface free energy of chitosan/hydroxypropyl-methylcellulose (HPMC) films plasticized with sorbitol. Mater. Sci. Eng..

[44] Dragostin O.M., Samal S.K., Lupascu F., Pânzariu A., Dubruel P., Lupascu D., Tuchiluș C., Vasile C., Profire L. (2015). Development and Characterization of Novel Films Based on Sulfonamide-Chitosan Derivatives for Potential Wound Dressing. Int. J. Mol. Sci..

[45] Van Oss C.J., Chaudhury M.K., Good R.J. (1988). Interfacial Lifshitz-van der Waals and polar interactions in macroscopic systems. Chem. Rev..

[46] Nwe N., Furuike T., Tamura H. (2009). The Mechanical and biological properties of chitosan scaffolds for tissue regeneration templates are significantly enhanced by chitosan from *Gongronella butleri*. Materials.

[47] Gethin G. (2007). The significance of surface pH in chronic wounds. Wounds UK.

[48] Menzies K.L., Jones L. (2010). The impact of contact angle on the biocompatibility of biomaterials. Optom. Vis. Sci..

[49] Zangi S., Hejazi I., Seyfi J., Hejazi E., Khonakdar H.A., Davachi S.M. (2016). Tuning cell adhesion on polymeric and nanocomposite surfaces: Role of topography versus superhydrophobicity. Mater. Sci. Eng.C.

